# Adjunctive Multicomponent Crystals of Two Anti-Tubercular Drugs with Pyridoxine

**DOI:** 10.3390/pharmaceutics18030297

**Published:** 2026-02-27

**Authors:** Tsebang A. Matlapeng, Theodor E. Geswindt, Roderick B. Walker, Vincent J. Smith

**Affiliations:** 1 Department of Chemistry, Rhodes University, Makhanda 6140, South Africa; g17m5217@campus.ru.ac.za (T.A.M.); t.geswindt@ru.ac.za (T.E.G.); 2 Faculty of Pharmacy, Rhodes University, Makhanda 6140, South Africa; 3 School of Pharmacy, Applied Sciences and Public Health, Robert Gordon University, Aberdeen AB10 7AQ, UK; 4 Department of Chemistry and Polymer Science, Stellenbosch University, Stellenbosch 7599, South Africa

**Keywords:** TB, cocrystallisation, salt, pyridoxine, 4-aminosalicylic acid, pyrazinecarboxylic acid, mechanochemistry, adjunctive cocrystal, multicomponent crystals

## Abstract

**Background/Objectives**: Cocrystallisation is a well-established path for altering the physicochemical properties and bioavailability of active pharmaceutical ingredients (APIs). A common side effect of anti-tubercular medicines is the depletion of group B vitamin reserves in TB patients. Co-administration of supplements such as pyridoxine (vitamin B_6_) during TB therapy may be used to ameliorate the harmful side effects of vitamin B_6_ deficiency. **Methods**: Mechanochemical grinding and solvent evaporation experiments using pyridoxine (PN) with 4-aminosalicylic acid (PAS) and separately with pyrazinecarboxylic acid (PCBA) were conducted. The bulk powder and crystal analysis was performed using FTIR, PXRD, DSC, TGA and SCXRD. **Results**: The isolation and characterization of two multicomponent salts containing pyridoxine, i.e., PN-PAS·H_2_O and PN-PCBA, were completed. Mechanochemistry is an efficient method for the preparation of cocrystals. **Conclusions**: The drug–vitamin combinations may be useful for the development of new treatment regimens with potentially improved therapeutic outcomes and reduced adverse effects.

## 1. Introduction

Tuberculosis (TB) is a bacterial infection caused by *Mycobacterium tuberculosis* that is the second most infectious disease after COVID-19. It is ranked amongst the top 10 leading causes of death worldwide, with an estimated 10.7 million people having contracted TB in 2024, while about 1.23 million TB-related deaths were reported over the same period [1]. TB is a treatable disease; however, it suffers from issues associated with drug resistance due to patient non-adherence to treatment regimens, health care providers prescribing the wrong treatment and the unavailability of medicines in some communities [1].

The first- and second-line therapies recommended by the WHO were introduced more than 50 years ago. The youngest member of the first-line quartet of drugs, consisting of rifampicin, isoniazid, ethambutol and pyrazinamide, was introduced in 1970 [1]. During this time, first-line medicines have been linked with issues that include rifampicin, isoniazid and pyrazinamide resistance, as well as isoniazid-induced pyridoxine deficiency, among others [2]. On the other hand, the second-line regimens are less effective, more toxic and are often requiring parenteral administration, which can cause significant patient discomfort [3].

The new drug ‘pipeline’ for TB is slow, and the process is costly with uncertain outcomes. The attrition rate of new medicines in preclinical and clinical development stages is high and ultimately leads to long periods between the registration of new medicinal therapies. Only two new drug candidates, bedaquiline and delamanid, have reached the market within the last decade (2014) [4,5,6]. These issues have led to the repurposing and reuse of active pharmaceutical ingredients (APIs) to define new treatment regimens.

Cocrystallisation (i.e., cocrystal formation) is a well-established vehicle for the repurposing and reuse of existing APIs through the preparation of unique crystalline materials, which consist of two or more components that are atoms, ions or molecules. Cocrystallisation is based on crystal engineering principles that are well suited to the solid-state modification of physicochemical properties [7,8,9,10]. When suitable, pharmaceutically acceptable coformers are chosen it may lead to multicomponent compounds that possess altered physicochemical properties as well as an improved pharmacological efficacy. The formation of pharmaceutical cocrystals or salts through cocrystallisation has previously been used to address some of the aforementioned issues with TB therapy. Multicomponent formation has been used to increase the chemical stability of para-amino salicylic acid against decarboxylation [11], to improve the oral bioavailability of quabodepistat [12], and to regulate the release of isoniazid [13], among other effects [11,12,13,14,15,16].

Mechanochemistry is defined as any chemical reaction induced by the direct absorption of mechanical energy [17]. These reactions are consistent with green chemistry owing to their reduced or non-existent solvent use, improved atom economy, involvement of catalysts, minimal energy input and minimal waste production, among other features [18]. Mechanochemistry is an efficient cocrystal screening or viability determination method that can be implemented to avoid multiple recrystallisations or sublimation attempts in search of a suitable solvent, to negate solubility issues, to prevent the exposure of starting materials to harsh conditions and to minimize the waiting times associated with numerous recrystallisation attempts [19,20,21].

**Scheme 1:**
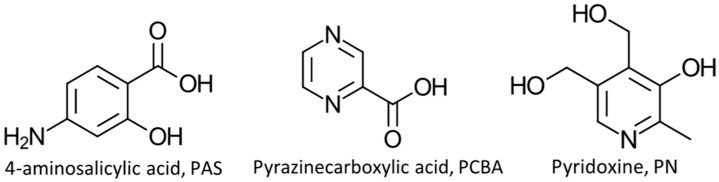
Molecular diagrams of PAS, PCBA, and PN.

Our choice of API is based on an adjunctive approach to the selection of coformers (Scheme 1), which means the coformers can potentially enhance the efficacy of the API [22]. The APIs selected for the study are 4-aminosalicylic acid (PAS), which is a second-line drug used in the treatment of multi-drug-resistant tuberculosis but has low solubility and is chemically unstable [23]; pyrazinecarboxylic acid (PCBA), which is the active metabolite of the first-line drug pyrazinamide and circumvents having to deal with pyrazinamide resistance [24]; and pyridoxine (PN), a B vitamin used to supplement vitamin B6 during TB therapy [25]. The aim of our investigation is the preparation and characterization of multicomponent crystals of the selected APIs.

## 2. Materials and Methods

### 2.1. Materials

PN, PAS, and PCBA were purchased from Sigma-Aldrich, Johannesburg, South Africa. Methanol (MeOH), ethyl acetate (EtOAc) and acetonitrile (MeCN) were purchased from Tag Solvent Products (Germiston, South Africa). Ultrapure water was produced using a RephiLe Direct-Pure Microsep^®^, (Sandton, South Africa) and a 10-inch 4-stage prefiltration kit comprised of a 10 µm SupaSpun DOE prefilter (Amazon Filters, Surrey, UK), an anti-scale conditioning cartridge, a 5 µm SupaCarb activated carbon filter, and a 0.5 µm SupaSpun II Absolute filter (Amazon Filters, Surrey, UK). The water was filtered through a 0.2 µm PES high flux capsule filter at the collection point before use, and the water quality was 18.2 MΩ·cm at 25 °C, with a total organic carbon (TOC) level of <2 ppb. All materials were used as received without any further purification.

### 2.2. Cocrystal Preparation

#### 2.2.1. Solution Crystallisation

Single crystals of PN-PAS·H_2_O (**1**) (1:1) were prepared by dissolving equimolar quantities of PN (0.065 mmol, 11.4 mg) and PAS (0.065 mmol, 10.4 mg) in a mixture of MeOH and EtOAc (ratio 1:3 *v*/*v*) at 45 °C to facilitate complete dissolution. The solution was left under ambient conditions (approximately 20 °C and 101.3 kPa) to slowly evaporate, producing diffraction-quality crystals after several days. Single crystals of PN-PCBA (**2**) (1:1) were prepared by dissolving equimolar quantities of PN (0.08 mmol, 13.6 mg) and PCBA (0.08 mmol, 10.4 mg) in a mixture of MeOH and MeCN (ratio 1:3 *v*/*v*) at 50 °C to facilitate complete dissolution. The solution was left at approximately 20 °C to slowly evaporate, producing diffraction-quality crystals.

#### 2.2.2. Mechanochemical Reactions (NG and LAG)

All products were prepared by grinding equimolar amounts of reactant APIs using a mechanical ball mill constructed from a Makita Jigsaw (Makita Corporation Japan, Anjo, Japan) fitted with a 2748.0 mm^3^ milling steel capsule and a single stainless-steel ball with a 4.5 mm diameter. Each grind was performed for 20 min at a frequency of 16 Hz. The milling capsule was securely clamped to the jigsaw blade via a custom U-bracket, while the steel capsule was fixed into the bracket at both ends using M4 bolts. The milling frequency was monitored in real time using an infrared beam sensor with a digital frequency counter. In the case of the liquid-assisted grinding (LAG) experiments, 15 μL of solvent was added to the reaction mixture prior to grinding. The LAG experiments were repeated four times, each time using a different solvent, viz., MeOH, EtOAc, MeCN and H_2_O. No solvent was added to the neat grind (NG) experiments.

### 2.3. Single-Crystal X-Ray Diffraction (SCXRD)

Single-crystal X-ray diffraction (SCXRD) was carried out using a Bruker D8 Venture diffractometer fitted with a PHOTON II CPAD detector (Bruker, Karlsruhe, Germany) and an Incoatec IμS 3.0 micro source combined with HELIOS optics. Mo-Kα radiation of wavelength 0.71073 Å was used for all data collections. Data was collected using ϕ- and ω-scans. An Oxford Cryosystems Cryostream 800 series (Oxford Cryosystems, Long Hanborough, UK) was used for temperature control at 100 K. Data reduction was carried out by means of a standard procedure using the Bruker software package SAINT V8.40B, and absorption corrections and correction of other systematic errors were performed using SADABS within APEX4 [26]. The structures were solved by direct methods using SHELXT 2014/5 [27] and refined using SHELXL 2016/6 [28]. X-Seed V4.20 [29] was used as the graphical interface for the SHELX program suite. All atoms were refined anisotropically based on well-behaved isotropic temperature factors, while hydrogen atoms were placed in idealized positions in a riding model, except when they were attached to heteroatoms; these were refined using observed peak heights in the difference Fourier map. The disorder of the aliphatic hydroxyl group observed in **1** was refined by summing the electron density for the two sites and calculating a percentage occupancy based on the electron density at each site. The site occupancy of the water molecule present in **1** is based on its proximity to the disordered aliphatic hydroxyl moiety.

### 2.4. Powder X-Ray Diffraction (PXRD)

Powder X-ray diffraction data were collected on a Bruker D2 phaser 2nd generation instrument (Bruker, Karlsruhe, Germany) fitted with Cu-Kα radiation source (λ = 1.54184 Å) and Lynxeye detector, with power settings of 30 kV and 10 mA. Data were collected over the 2θ range 5–40°, with a step size of 0.04°.

### 2.5. Differential Scanning Calorimetry (DSC)

DSC experiments were performed on a Discovery DSC 250 (TA instruments, New Castle, DE, USA). Samples were placed in sealed Tzero aluminium pans and heated at a rate of 10 K/min, with a nitrogen purge flow rate of 50 mL/min.

### 2.6. Thermogravimetric Analysis (TGA)

TGA was carried out on a PerkinElmer TGA 4000 instrument (PerkinElmer, Waltham, MA, USA). Samples were placed in a 180 μL ceramic pan and heated in a ceramic furnace at 10 K/min, with a nitrogen purge flow rate of 20 mL/min.

### 2.7. Fourier Transform Infrared-Attenuated Total Reflectance Spectroscopy (ATR-FTIR)

FTIR spectra were recorded using a PerkinElmer Spectrum TWO FTIR instrument (PerkinElmer, Waltham, MA, USA) fitted with a universal attenuated total reflectance accessory. The collection window was set to 4000–400 cm^−1^, using 32 scans at a scan speed of 0.2 cm^−1^/s. The samples prepared by mechanochemical milling were scanned without further processing, while single crystals were dried on filter paper prior to crushing and scanning.

## 3. Results and Discussion

The investigation commenced with a viability study involving the milling of pairs of APIs in equimolar quantities under neat (NG) and liquid-assisted grinding (LAG) conditions. The LAG solvents were either polar protic or polar aprotic. The polar protic solvents used were water and methanol, while the polar aprotic solvents used were ethyl acetate and acetonitrile. Henceforth, the products obtained from NG will be denoted **1***a* and **2***a*, and the LAG products will be denoted **1***b* and **2***b* (acetonitrile), **1***c* and **2***c* (ethyl acetate), **1***d* and **2***d* (methanol), and **1***e* and **2***e* (water). The resulting products were characterized by PXRD, DSC, FTIR, and TGA, to establish the multicomponent phase formation. When new phases (i.e., multicomponent phases) were detected, recrystallisation experiments were carried out to obtain single crystals suitable for single-crystal X-ray diffraction. We commence with the single-crystal study first and report on the viability study last.

### 3.1. Crystal Structure Analysis

The two pyridoxine salts, **1** and **2**, were recrystallised from a mixture of methanol and ethyl acetate in the case of **1**, and methanol and acetonitrile in the case of **2**. Crystal data for both salts are summarized in Table S1 (Supplementary Materials). Salt formation was established based on changes in the individual bond lengths of the carboxylate moieties of PAS and PCBA, while electron density peaks near the nitrogen atom of the PN molecule, located in the difference Fourier map, were refined as hydrogen atoms.

**Figure 1:**
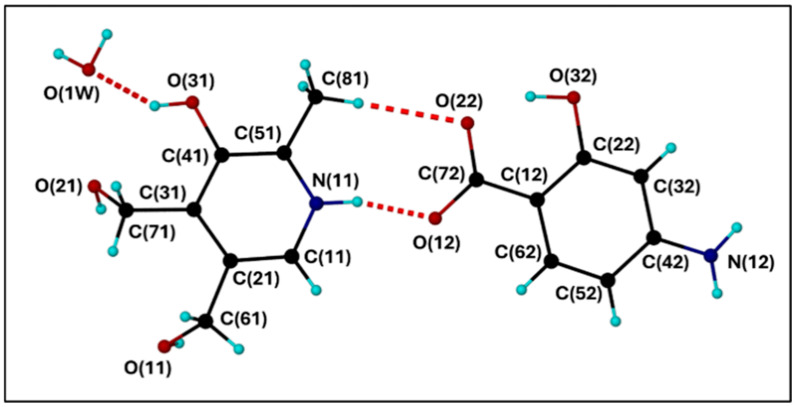
The labelled asymmetric unit of **1**, containing a molecule each of PN and PAS and a water molecule with partial occupancy. Hydrogen bonds are indicated by the red dashed lines.

Crystallising in the monoclinic space group *P*2_1_/*c*, the asymmetric unit of **1** (Figure 1) consists of a single molecule of PAS, a molecule of PN and a single water molecule with partial occupancy (s.o.f. = 0.63). The methyl hydroxy group located at the 3-position of the PN ring is disordered over two positions, where the methyl hydroxy group disorder is related to its proximity to the included water molecule. In the absence of the water molecule from the structure, the disordered methyl hydroxy group is co-planar to the pyridine ring of PN. When the water molecule is present, the methyl hydroxy group is rotated out of the plane of the ring, almost perpendicular to the mean plane of the ring. Therefore, the water molecule and the methyl hydroxy group have the same site occupancy value of 0.63 (or 63%), while in the absence of the water molecule the methyl hydroxy group has an occupancy of 0.37 (or 37%), as shown in Figure S1 (Supplementary Materials). PN and PAS interact via a charge assisted N-H⋯O hydrogen bond involving the protonated pyridine nitrogen atom (N11) and the deprotonated carboxylate oxygen atom belonging to PAS (O12). The interaction is further supported by a weak C-H⋯O interaction between a neighbouring methyl group (C81) of PN and the carboxylate oxygen atom of PAS (atom O22).

**Figure 2:**
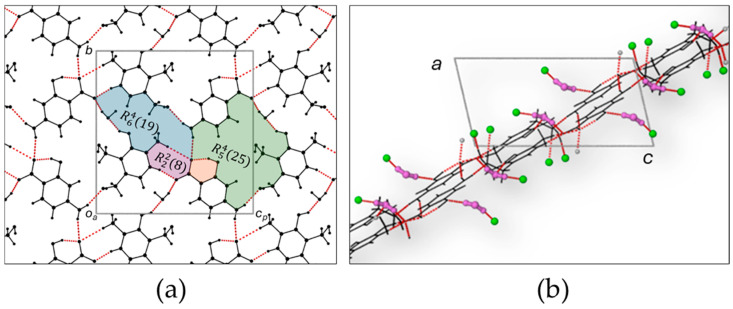
(**a**) The *bc* plane depicting the intricate hydrogen bonding network and motifs. (**b**) The view down the *b* axis showing how consecutive layers are linked via hydrogen bonds. (Purple = water molecules, green = oxygen atoms belonging to molecules in layers above and below). Hydrogen bonds are indicated by the red dashed lines.

The packing arrangement of **1**, when viewed down the *a* axis (onto the *bc* plane), shows a layer consisting of hydrogen-bonded PN and PAS molecules in an extensive network that contains three intermolecular hydrogen-bonded ring motifs. The first ring consists of five donor atoms and four acceptor atoms, with a total of 25 atoms completing the ring (the graph set descriptor is $R_{5}^{4}$(25)); meanwhile, the second ring involves six donor atoms and four acceptor atoms, with a total of 19 atoms completing the ring. Its graph set descriptor is $R_{6}^{4}$(19), as seen in Figure 2a [30,31]. The third ring contains two donors, two acceptors, and involves eight atoms ($R_{2}^{2}$(8)). Successive layers are hydrogen bonded to each other via the interstitial water molecules and methyl hydroxy moieties, which hydrogen bond to layers above and below (Figure 2b).

**Figure 3:**
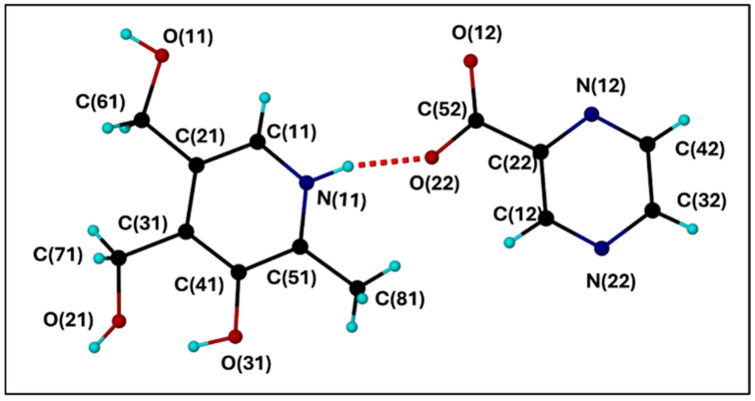
The labelled asymmetric unit of **2**, containing a molecule each of PN and PCBA. Hydrogen bonds are indicated by the red dashed lines.

The asymmetric unit of **2** comprises a molecule each of PN and PCBA, which are hydrogen bonded to each other via a charge-assisted N-H⋯O interaction, as depicted in Figure 3. As with **1**, PN and PBCA form a hydrogen-bonded unit that is part of a larger hydrogen-bonded network. One of the oxygen atoms (O12) of the carboxylate moiety of PCBA is bifurcated, forming two O-H⋯O hydrogen bonds to the methyl hydroxy moiety of two different PN molecules (O11 and O21).

**Figure 4:**
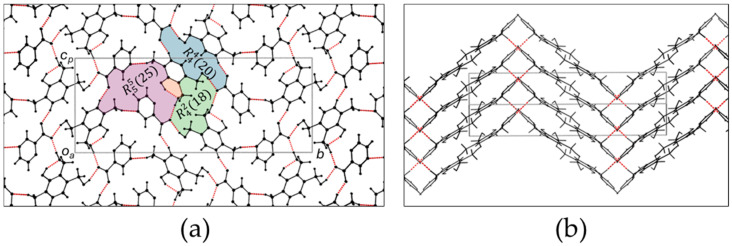
(**a**) The *bc* plane depicting the hydrogen-bonding network and hydrogen-bonding motifs. (**b**) View along the diagonal showing the zigzag arrangement of layers. Consecutive layers are hydrogen bonded to each other. Hydrogen bonds are indicated by the red dashed lines.

The O11-H61⋯O12 and the N11-H11n⋯O22 hydrogen bonding interactions are repeated through a centre of inversion, halfway along the *b* axis, forming a centrosymmetric ring consisting of 20 atoms, four H-bond donor atoms and four H-bond acceptor atoms, involving two PN molecules and two PCBA molecules. The graph set descriptor for this ring is $R_{4}^{4}(20)$ and is highlighted in blue in Figure 4a [30,31]. The second hydrogen-bonded ring, at the centre of the cell, is formed by a different set of PN and PCBA molecules. The bifurcated carboxylate atom (O12) hydrogen bonds with two different PN molecules through the oxygen atoms of the methyl hydroxy moieties located on the different molecules (O11-H61⋯O12 and O21-H71⋯O12), shaded green in Figure 4a. The hydrogen bonding is repeated through the centre of inversion at the centre of the cell, forming a ring with graph set descriptor $R_{4}^{2}(18)$. The third hydrogen-bonded intermolecular ring consists of five donors and acceptors and involves 25 atoms to complete the ring. The three hydrogen-bonded rings share atoms and molecules and combine to form corrugated sheets that span the crystal parallel to the *c* axis. The sheets are hydrogen bonded to each other by a single C81-H81c⋯N12 interaction, where the hydrogen-bond donor and acceptors are in different layers, as seen in Figure 4b.

**Figure 5:**
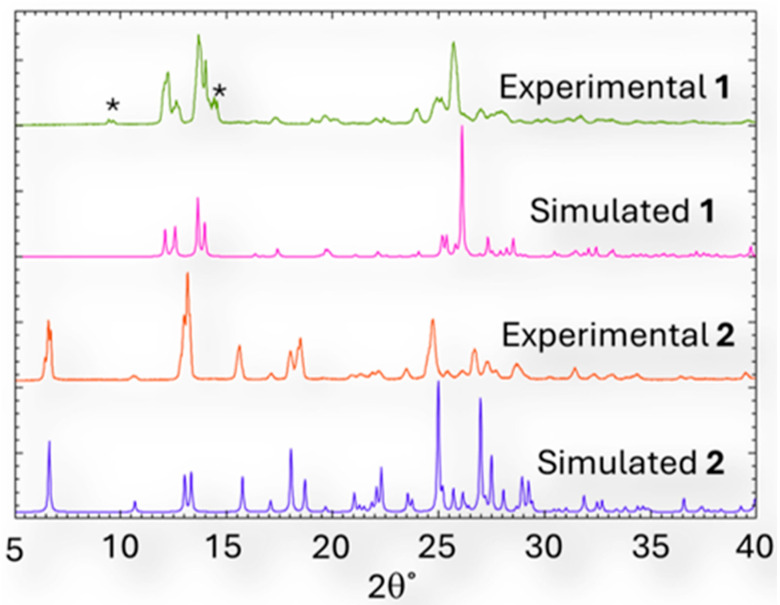
Comparison of the experimental and simulated PXRD profiles for compounds **1** and **2**. The asterisks “*” in the experimental profile of **1** indicate peaks related to unreacted starting material.

Diffractograms of the crystalline batches of **1** and **2** were compared to the simulated PXRD profiles of **1** and **2**. An excellent correlation exists between the experimental and simulated diffractograms, even though the experimental profile of **1** shows evidence of unreacted starting materials (Figure S2a,b, Supplementary Materials), as indicated by the asterisks in Figure 5.

### 3.2. Milling Experiments

After milling for approximately twenty minutes, the products of the mechanochemical experiments were analysed using PXRD, DSC, TGA and FTIR. The thermograms of the reactants were used as reference thermograms and used to compare the thermograms of the NG and LAG products.

**Figure 6:**
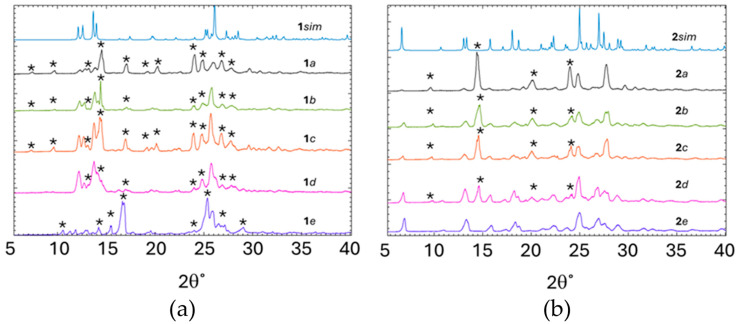
PXRD profiles of the simulated and mechanochemical preparations of: (**a**) **1** and (**b**) **2**. The asterisks “*” in the experimental profile of **1** indicate peaks related to unreacted starting material.

The PXRD profiles of the mechanochemically prepared samples (**1***a*, **1***b*, **1***c*, **1***d*, and **1***e*) were compared to the simulated profile **1***sim*, as seen in Figure 6a. The PXRD profiles of **1***a*, **1***b*, **1***c*, and **1***d* correlate poorly with the profile of **1***sim*, owing to the presence of several peaks that belong to unreacted starting materials (PN and PAS). These peaks are indicated in Figure 6a with an asterisk. Moreover, **1***a*, **1***b*, and **1***d* correlate well with each other, suggesting that the mechanochemical preparation of the salt is less favourable than the preparation by solution recrystallisation. The profile of **1***e* does not match any of the other profiles and the mismatch can be attributed to the presence of the decomposition products of PAS when ground with water and the presence of reactants [16,32].

While the PXRD profiles of **1***a*, **1***b*, **1***c*, **1***d*, and **1***e* were a poor correlation to the profile of **1***sim*, the PXRD profiles for **2***a*, **2***b*, **2***c*, and **2***d* correlate well with the profile of **2***sim*. However, there are some additional peaks which correspond to the unreacted starting materials (PN and PCBA), indicated with an asterisk (Figure 6b). The only exception seems to be **2***e*; the LAG experiment performed using water as solvent has no obvious peaks that belong to the unreacted starting material and thus, implies a higher level of conversion when using water as the solvent during LAG and is in stark contrast to **1***e*, which appears to have decomposed.

**Figure 7:**
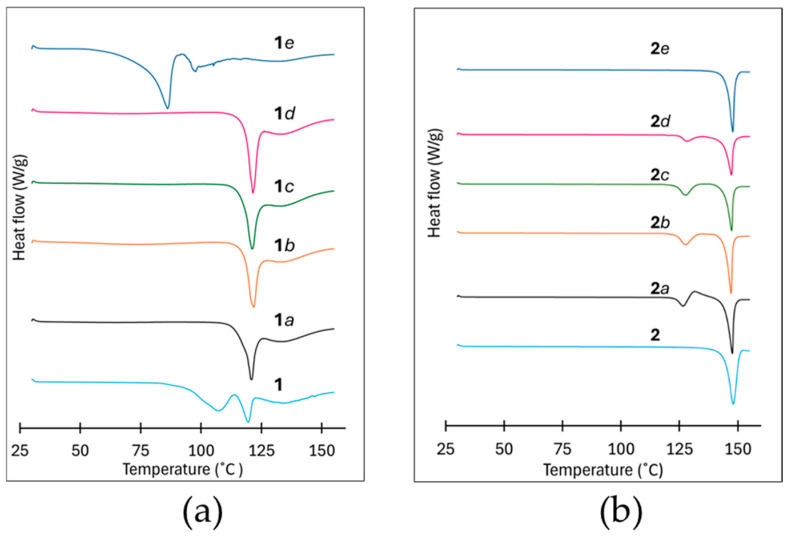
DSC thermograms of the milling samples and crystalline products of: (**a**) **1** and (**b**) **2**.

The DSC thermograms for **1***a*, **1***b*, **1***c*, and **1***d* each exhibit two endotherms: a broad shallow endotherm in the range of 50–90 °C, and a second sharp endotherm in the range of 112–122 °C. The first set of endotherms correlate well with the observed weight loss in the range of 45 to 80 °C on TGA, and is probably the loss of solvent used during the milling process. The second set of endotherms are the melting endotherms occurring in a lower temperature range than the melting endotherms of the starting materials (see Figure S3, Supplementary Materials), consistent with salt formation, since it coincides with the melting endotherm of **1**. However, the thermogram for **1***e* has a single large endotherm in the 65–90 °C range that is immediately followed by the onset of decomposition, as seen in Figure 7a.

These thermal events are also observed in the TGA thermograms, a mass loss in the range of 65–90 °C followed by the onset of decomposition, as depicted in Figure S4 of the Supplementary Materials. Based on the work reported by Jivani et al. [32] and Perlovich et al. [16], we hypothesize that PAS, when exposed to moisture and heat (during grinding), may undergo decomposition [16,32].

Similarly, the thermograms for **2***a*, **2***b*, **2***c* and **2***d* exhibit two thermal events (endotherms), as seen in Figure 7b. The first endotherm, in the range of 122–129 °C, is probably due to the melting of unreacted starting materials remaining after milling, whereas the second sharp endotherm in the range of 140–148 °C is likely the melting endotherm. This was confirmed using temperature-cycled experiments followed by PXRD experiments of the products after temperature cycling, as seen in Figure S5a,b of the Supplementary Materials. The thermogram of **2***e* exhibits a single endotherm in the range of 140–148 °C, which coincides with the melting endotherm of **2**. None of the samples show any evidence of solvent loss during thermogravimetric analysis (Figure S6, Supplementary Materials). All melting point onset and peak temperatures are reported in Table 1.

**Figure 8:**
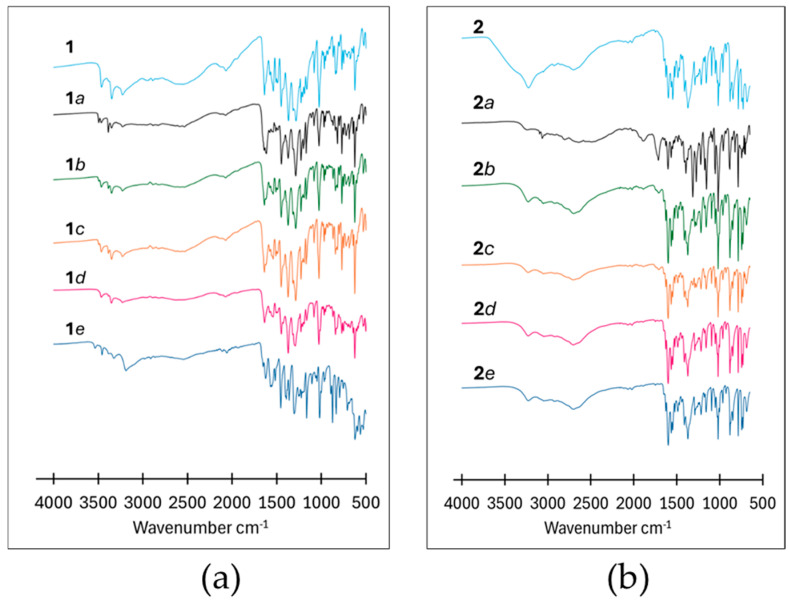
FTIR spectra of the crystalline and milling products of: (**a**) **1** and (**b**) **2**.

The FTIR spectrum of PAS is characterized by two amine bands *ν*(N-H) at 3494 and 3387 cm^−1^, a carbonyl stretch *ν*(C=O) at 1634 cm^−1^ and a broad *ν*(OH) band between 3095 and 2452 cm^−1^, while the spectrum of PN is characterized by a hydroxyl peak *ν*(OH) at 3278 cm^−1^, an aromatic amine band *ν*(C-N) centred at 1345 cm^−1^ and a secondary alcohol band *ν*(C-O) centred at 1067 cm^−1^, as seen in Figure S7 of the Supplementary Materials. The amine bands belonging to PAS are retained in the spectra of **1***a*, **1***b*, **1***c* and **1***d* with peak positions shifting to lower wavenumbers, as seen in Figure 8a. However, these peaks are split, doubling up on the number of peaks in the amine region, which is attributed to the presence of unreacted starting material. The hydroxyl and secondary alcohol bands present in the spectrum of PN are shifted relative to their position in the spectrum of pure PN (Figure S7). Two new broad bands are observed in the ranges of 1900–2200 cm^−1^ and 2200–2800 cm^−1^.

The formation of these broad bands in this region is indicative of strong hydrogen bond formation between the sample components PAS and PN [33]. Apart from **1***e*, the bands are present in the spectra of all the milled samples. Table 2 provides a summary of the shifts in wavenumbers for the pertinent functional groups.

The spectrum for pure PCBA contains two broad characteristic bands centred at 2214 cm^−1^ and 1884 cm^−1^ due to intermolecular hydrogen bonding between the carboxylic acid moiety and the aromatic nitrogen atom *ν*(O-H_acid_⋯N_aromatic_), as well as a carbonyl stretch at 1715 cm^−1^ [33], as seen in Figure S7 of the Supplementary Materials. The milled samples possess several aspects of both starting materials which contribute substantially to the final spectra, especially for **2***a*, **2***b*, and **2***c*. The two broad bands centred at 2214 cm^−1^ and 1884 cm^−1^ ascribed to PCBA become less prominent in all the LAG spectra. The spectrum for **2***a* exhibits characteristic peaks for unreacted PCBA (at 3065, 2496, 1887 and 1716 cm^−1^); however, these occur at slightly shifted wavenumbers compared to the pure PCBA, as seen in Figure 8b.

## 4. Conclusions

Mechanochemistry proved to be an efficient route for the rapid preparation of multicomponent forms; however, careful optimization remains essential for interpreting viability studies. Phase-verification analyses revealed the presence of unreacted starting materials, underscoring the need to employ complementary characterization techniques to confirm phase formation. For example, PXRD diffractograms clearly indicated residual starting materials (marked with asterisks), whereas the DSC data confirmed such residues only for compound **2**. Despite several positive outcomes, solvent selection continues to be a critical factor in identifying conditions conducive to crystallisation. Even a limited set of protic and aprotic solvents enabled systematic evaluation of solvent effects on the reaction outcomes.

- Water proved unsuitable for all systems: PAS decomposed under both LAG and recrystallisation conditions, while the PN-PCBA salt showed little to no unreacted material, exhibiting a single DSC event and a clean PXRD pattern.
- Both pyridoxine salts were obtained using the LAG and NG methods, providing greener and operationally simple synthetic alternatives.
- The remaining solvents (MeOH, EtOAc and MeCN) yielded crystalline pyridoxine salts suitable for single-crystal structure determination.

The ability to obtain crystalline products remains central to comprehensive solid-state characterization. Importantly, the mechanochemical approach substantially reduced solvent screening requirements, solvent consumption and the extended time frames typically associated with conventional recrystallisation workflows.

## Figures and Tables

**Table 1:** Melting point onset, peak temperatures and enthalpies for **1** and **2**.

Samples	T_onset_ (°C)	T_peak_ (°C)	Enthalpy (J/g)
**1**	110.6	120.0	21.4
**1** *a*	112.3	120.8	57.5
**1** *b*	113.3	121.0	68.6
**1** *c*	115.2	121.9	59.5
**1** *d*	115.8	121.6	74.1
**1** *e*	77.6	decomp.	
**2**	143.9	149.0	168.5
**2** *a*	141.9	147.4	138.5
**2** *b*	140.6	147.1	105.4
**2** *c*	141.1	146.9	107.8
**2** *d*	140.6	147.3	109.0
**2** *e*	142.5	147.6	140.0

**Table 2:** Common stretching/bending frequencies (cm^−1^) for all preparations of **1** and **2**.

Vibrational Modes	PAS	PN	1
*ν*(OH)	-	3278	3234
*ν*(NH_2_)	3494, 3387	-	3499, 3391
*ν*(C=O)	1615	-	1638
*ν*(C-O)	1286	-	1286
*ν*(C-OH)	-	1019	1025
	**1** *a*	**1** *b*	**1** *c*
*ν*(OH)	3237	3238	3237
*ν*(NH_2_)	3472, 3357	3472, 3356	3470, 3354
*ν*(C=O)	1622	1639	1640
*ν*(C-O)	1289	1289	1289
*ν*(C-OH)	1026	1027	1027
	**1** *d*	**1** *e*	
*ν*(OH)	3235	3334	
*ν*(NH_2_)	3472, 3358	3462, 3358	
*ν*(C=O)	1638	1625	
*ν*(C-O)	1293	1253	
*ν*(C-OH)	1028	1020	
	PCBA	PN	**2**
*ν*(OH)	-	3278	3240
*ν*(C=O)	1714	-	1607
*ν*(C-O)	1272	-	1290
*ν*(C-OH)	-	1019	1018
	**2** *a*	**2** *b*	**2** *c*
*ν*(OH)	3258	3248	3253
*ν*(C=O)	1716	1602	1603
*ν*(C-O)	1274	1291	1291
*ν*(C-OH)	1019	1019	1019
	**2** *d*	**2** *e*	
*ν*(OH)	3246	3244	
*ν*(C=O)	1603	1601	
*ν*(C-O)	1291	1290	
*ν*(C-OH)	1019	1019	

## Data Availability

The original contributions presented in this study are included in the article/Supplementary Material. Further inquiries can be directed to the corresponding authors.

## Supplementary Materials

The following supporting information can be downloaded at: https://www.mdpi.com/article/10.3390/pharmaceutics18030297/s1. Table S1: Crystal data and structure refinement for **1** and **2**; Figure S1: Observed disorder of the aliphatic hydroxyl group and the water molecule in the structure of **1** (PN-PAS·H_2_O, the colours represent the corresponding site occupancies); Figure S2: (a) The simulated and experimental PXRD profiles of **1** along with the profiles of the starting materials for **1**; (b) The simulated and experimental PXRD profiles of **2** along with the profiles of the starting materials for **2**; Figure S3: DSC thermograms of the starting materials; Figure S4: TGA thermograms for the crystalline and milling products of **1** (PN-PCBA); Figure S5: (a) DSC thermogram of **2**a showing disappearance of the first endotherm after temperature cycling; (b) PXRD profiles of **2**a and **2**c before and after temperature cycling; Figure S6: TGA thermograms for the milling products of **2** (PN-PCBA); Figure S7: FTIR spectra of the starting materials.

## Abbreviations

The following abbreviations are used in this manuscript:

API	Active pharmaceutical ingredient
NG	Neat grind
LAG	Liquid-assisted grind
