# Supplementary material for: Adjunctive Multicomponent Crystals of Two Anti-Tubercular Drugs with Pyridoxine
Source: Pharmaceutics. 2026 Feb 27;18(3):297. doi: 10.3390/pharmaceutics18030297 (PMC13029089; doi:10.3390/pharmaceutics18030297)
Supplement: Supplementary file 1 [file pharmaceutics-18-00297-s001.zip › pharmaceutics-4120314-supplementary.pdf]

Adjunctive Multicomponent Crystals of Two Anti-Tubercular Drugs with Pyridoxine

Tsebang A. Matlapeng [1], Theodor E. Geswindt [1], Roderick B. Walker [2,3,*] and Vincent J. Smith [1,4,*]

Table of contents

Table S1: Crystal data and structure refinement for **1** and **2**........ 1

Figure S1: Observed disorder of the aliphatic hydroxyl group and the water molecule in the structure of **1** (PN-PAS·H_2O, the colours represent the corresponding site occupancies)........ 1
Figure S2a: The simulated and experimental PXRD profiles of **1** along with the profiles of the starting materials for **1**........ 2
Figure S2b: The simulated and experimental PXRD profiles of **2** along with the profiles of the starting materials for **2**........ 4
Figure S3: DSC thermograms of the starting materials. 3
Figure S4: TGA thermograms for the crystalline and milling products of **1** (PN-PCBA). 3
Figure S5a: DSC thermogram of **2***a* showing disappearance of the first endotherm after temperature cycling. 4
Figure S5b: PXRD profiles of **2***a* and **2***c* before and after temperature cycling........ 4
Figure S6: TGA thermograms for the milling products of **2** (PN-PCBA). 5
Figure S7: FTIR spectra of the starting materials........ 5

Table S1: Crystal data and structure refinement for 1 and 2.

**Data collection and refinement parameters**	**1**	**2**
CCDC no.	2517508	2517506
Formula unit	$(C_8H_{12}NO_3)$ $(C_7H_6NO_3)$ $0.63(H_2O)$	$(C_8H_{12}NO_3)$ $(C_5H_3N_2O_2)$
Formula mass (g mol^{-1})	333.66	293.28
Crystal system	monoclinic	monoclinic
Space group	$P2_1/c$ (No. 14)	$P2_1/n$ (No. 14)
a/Å	7.2978(3)	4.5766(2)
b/Å	14.5994(6)	26.5210(12)
c/Å	14.4328(6)	10.5787(5)
α/°	90	90
β/°	103.142(2)	90.855(2)
γ/°	90	90
Volume (Å^3)	1497.45(11)	1283.86(10)
Z	4	4
D_{calc} (g cm^{-3})	1.480	1.517
F (000)	705	616
μ (MoKα) (mm^{-1})	0.117	0.118
Crystal size (mm^3)	0.27 x 0.36 x 0.39	0.10 x 0.14 x 0.17
Temperature (K)	100	100
Range scanned ⊚ (°)	2.0 - 35.8	2.1- 28.3
Index ranges	-12: 10 ; -23: 23 ; -23: 23	-5: 6 ; -35: 35 ; -14: 14
Total number of reflections collected	100239	46424
Number of unique reflections	6936	3180
Number of reflections with $I > 2\sigma$ (I)	5510	2677
Number of least-squares parameters	270	208
R_{int}	0.049	0.051
S	1.04	1.04
R_1 ($I > 2\sigma$ (I))	0.0401	0.0342
wR_2	0.1193	0.0904
Weighting scheme parameters	a = 0.0618, b = 0.3462	a = 0.0387, b = 0.7130
$(\Delta/\sigma)_{mean}$	0.00	0.00
$\Delta\rho$ excursions (e Å^{-3})	-0.30, 0.50	-0.25, 0.31

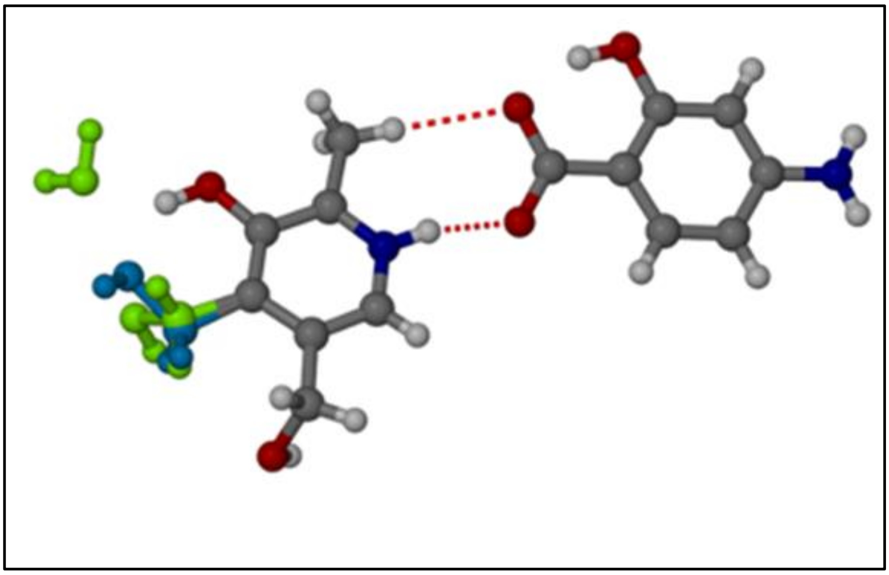

Figure S1: Observed disorder of the aliphatic hydroxyl group and the water molecule in the structure of **1** (PN-PAS·H_2O, the colours represent the corresponding site occupancies).

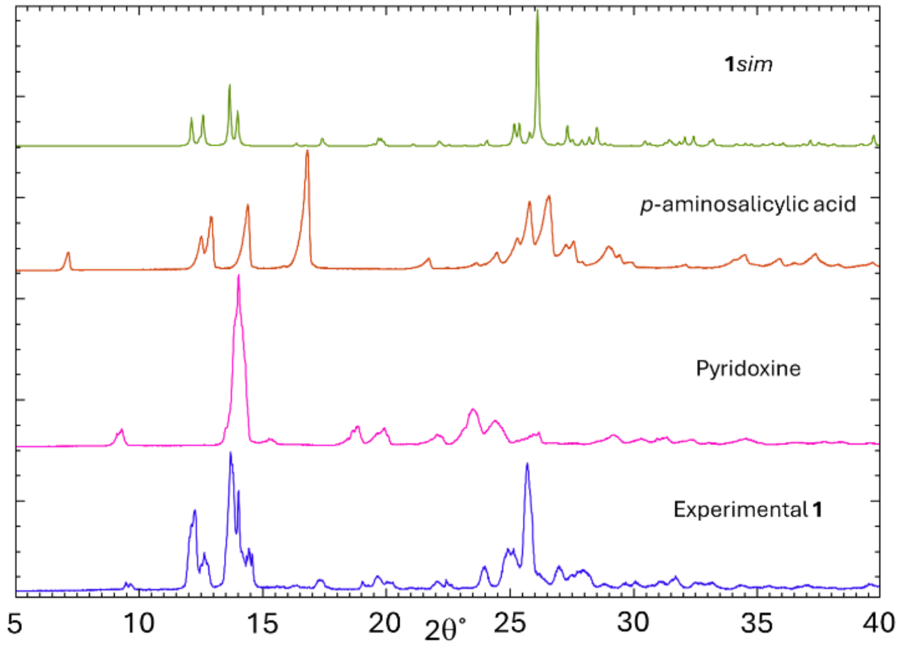

Figure S2a: The simulated and experimental PXRD profiles of **1** along with the profiles of the starting materials for **1**.

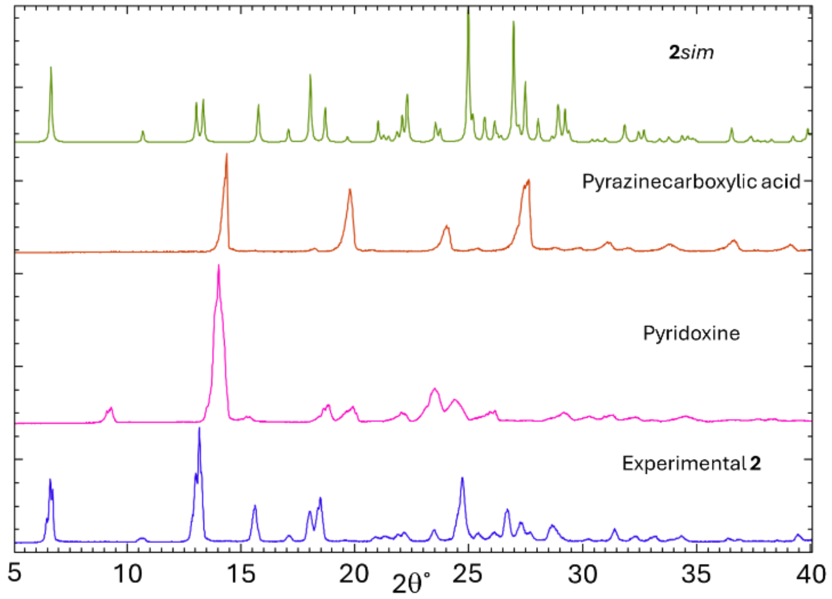

Figure S2b: The simulated and experimental PXRD profiles of **2** along with the profiles of the starting materials for **2**.

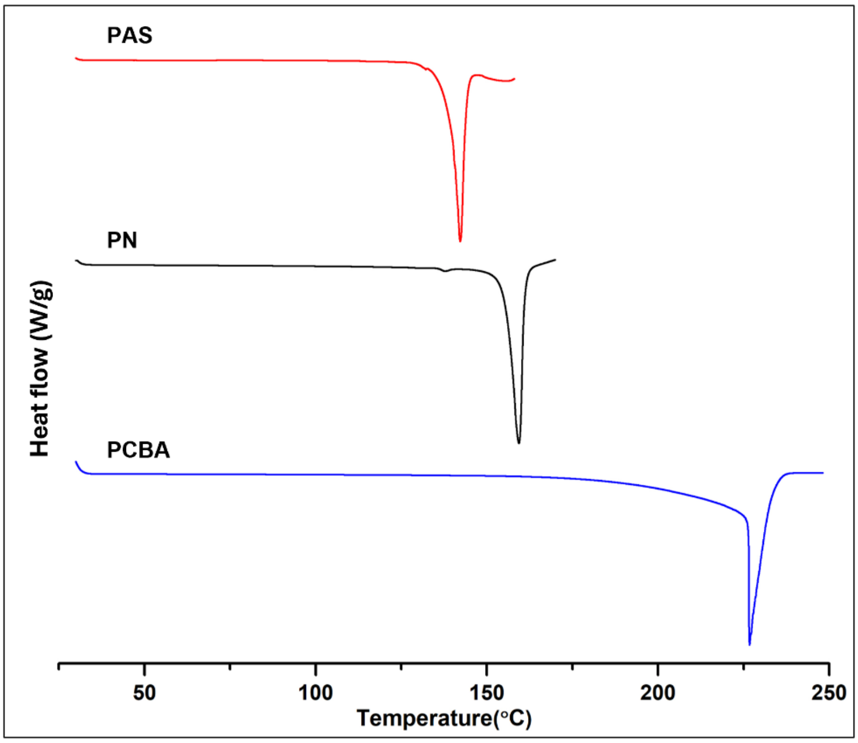

Figure S3: DSC thermograms of the starting materials.

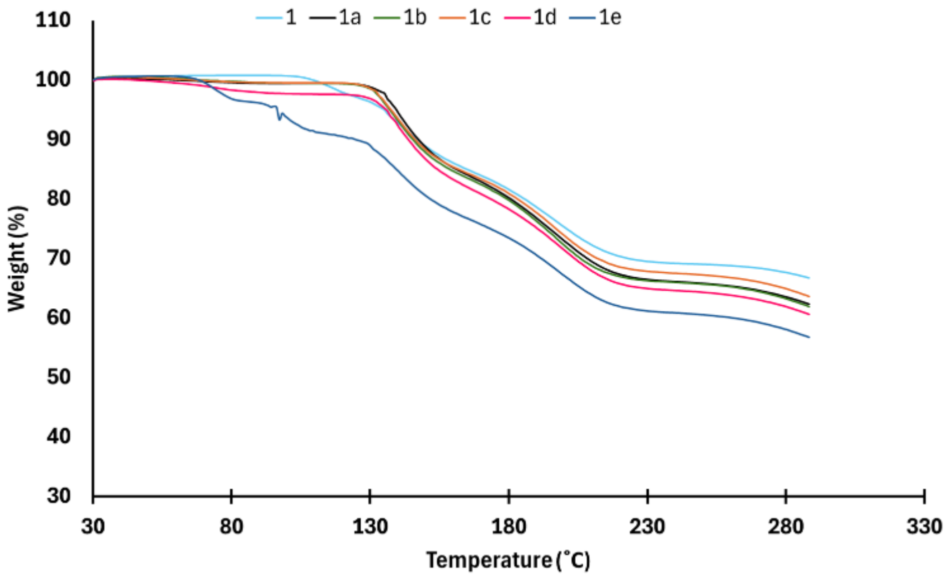

Figure S4: TGA thermograms for the crystalline and milling products of **1** (PN-PCBA).

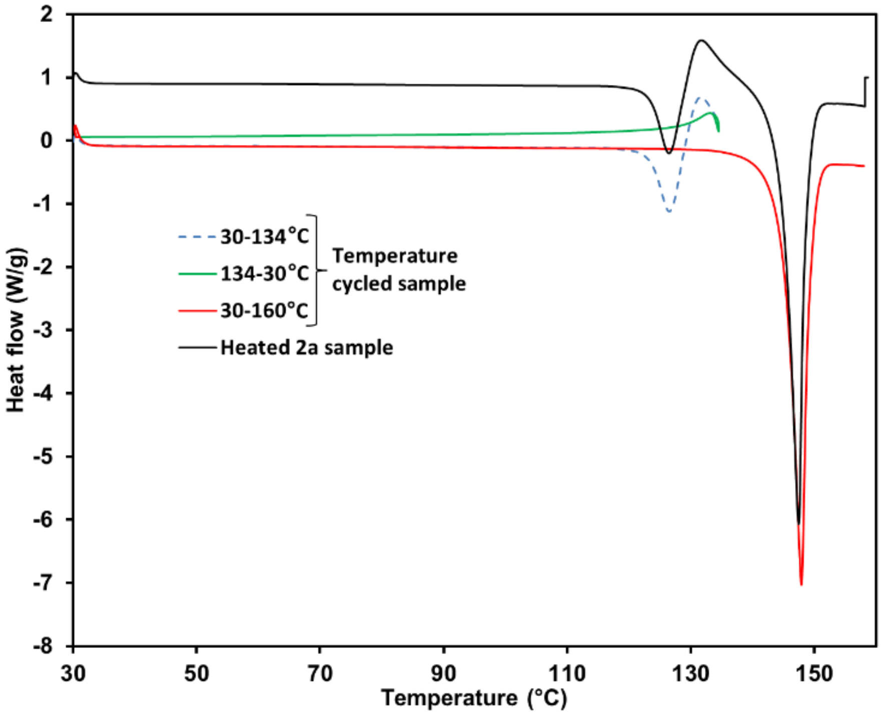

Figure S5a: DSC thermogram of **2***a* showing disappearance of the first endotherm after temperature cycling.

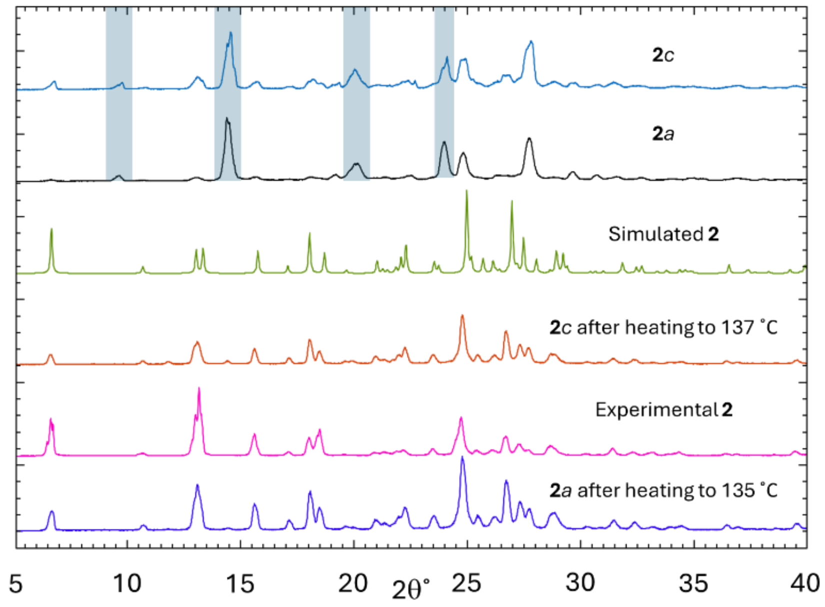

Figure S5b: PXRD profiles of **2***a* and **2***c* before and after temperature cycling.

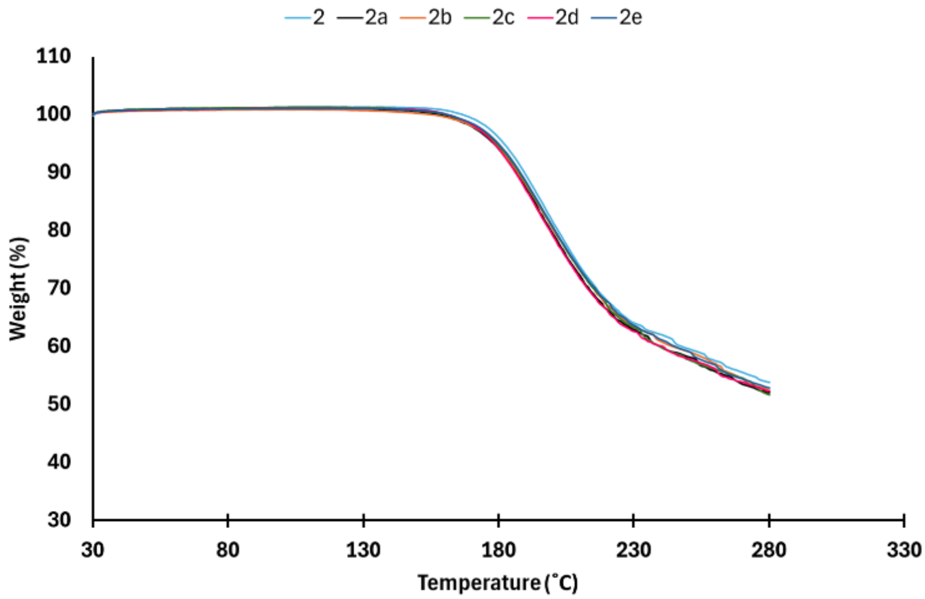

Figure S6: TGA thermograms for the milling products of **2** (PN-PCBA).

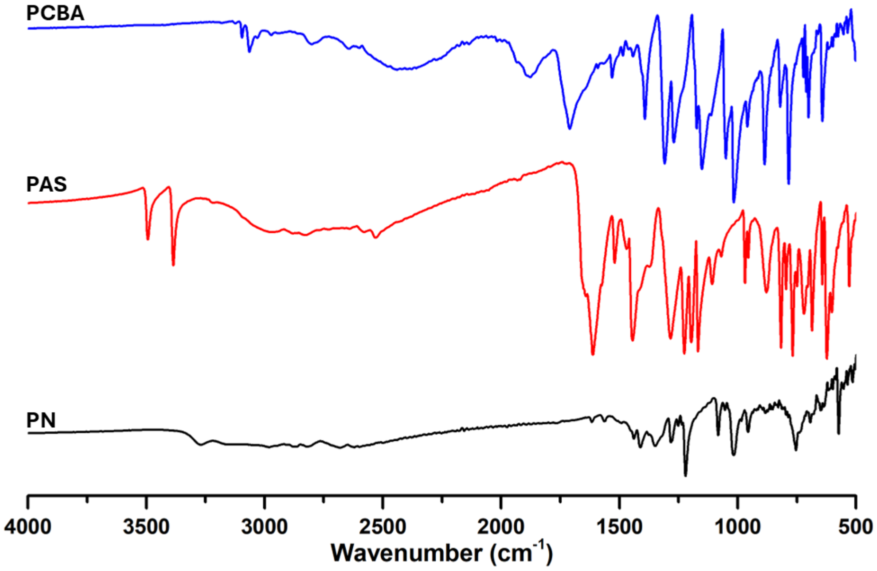

Figure S7: FTIR spectra of the starting materials.
